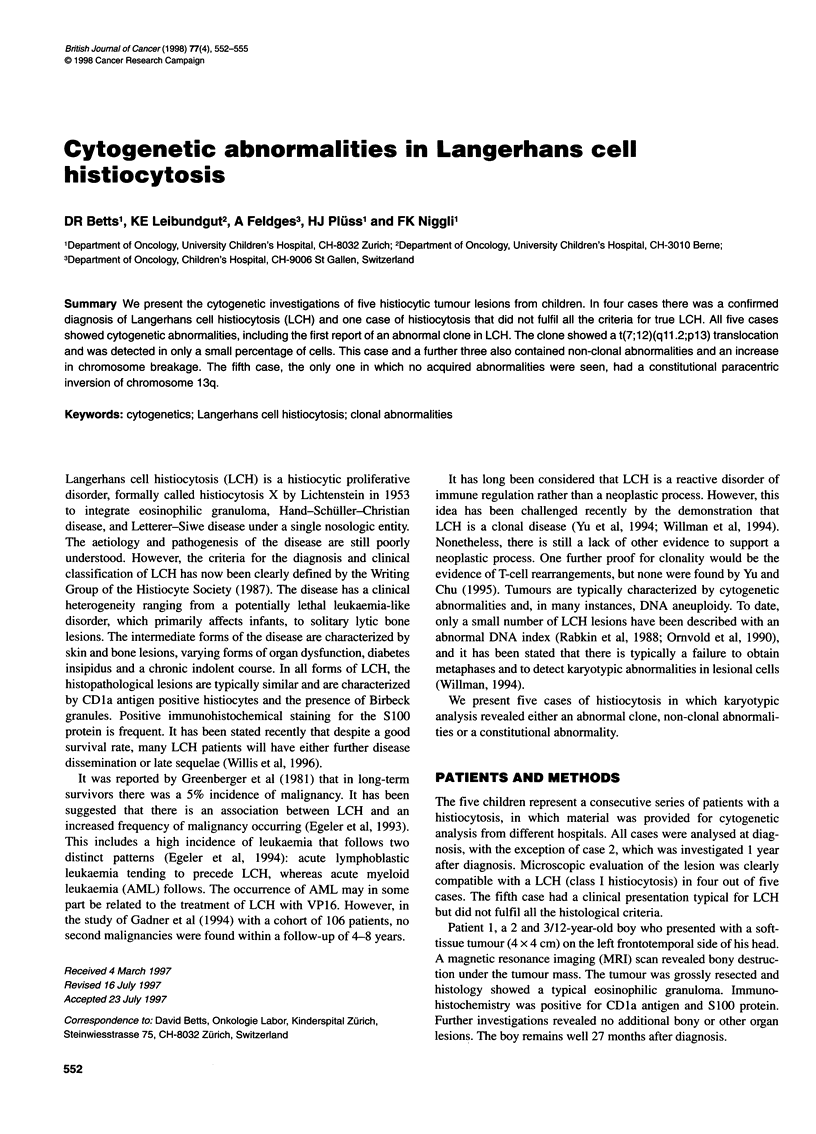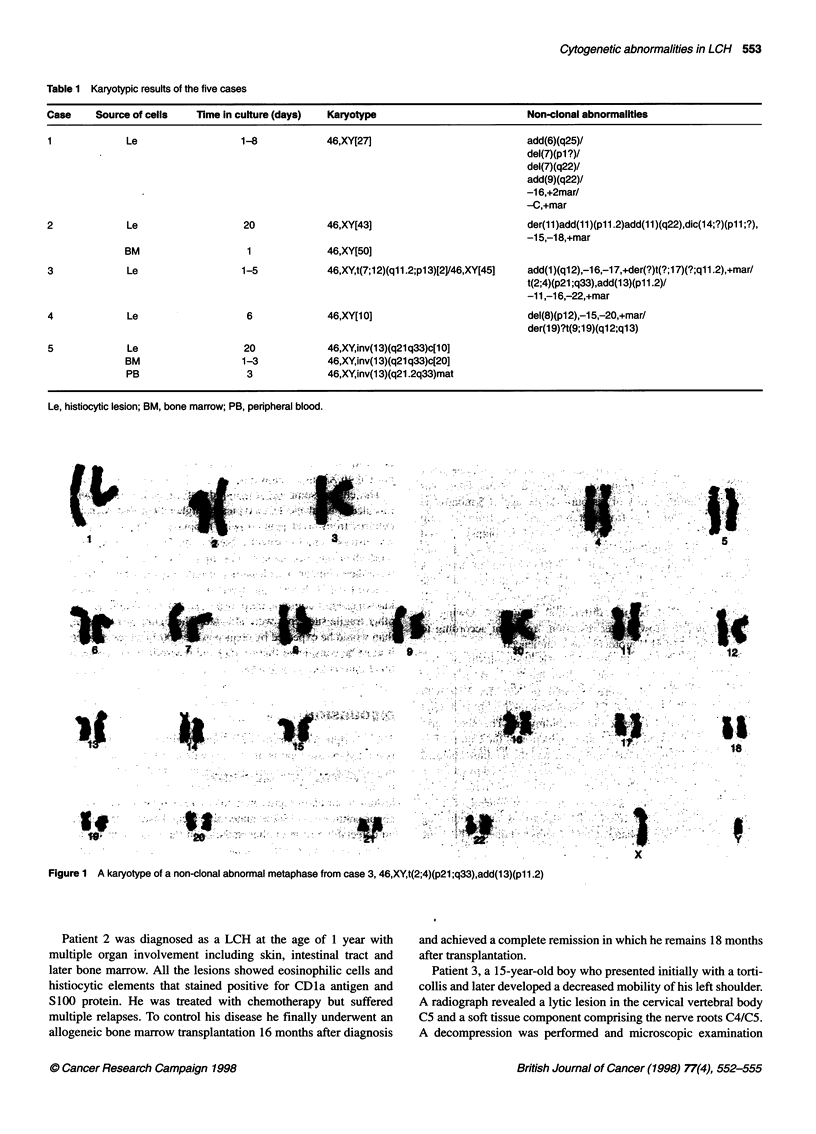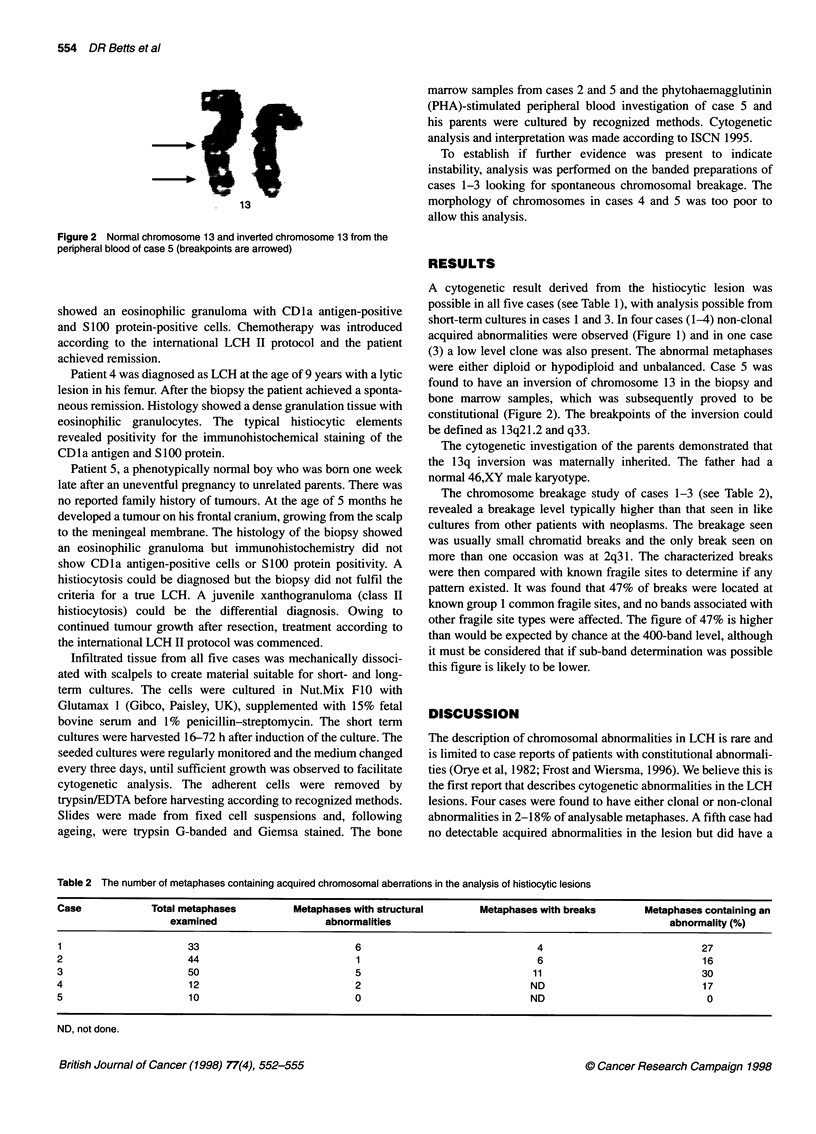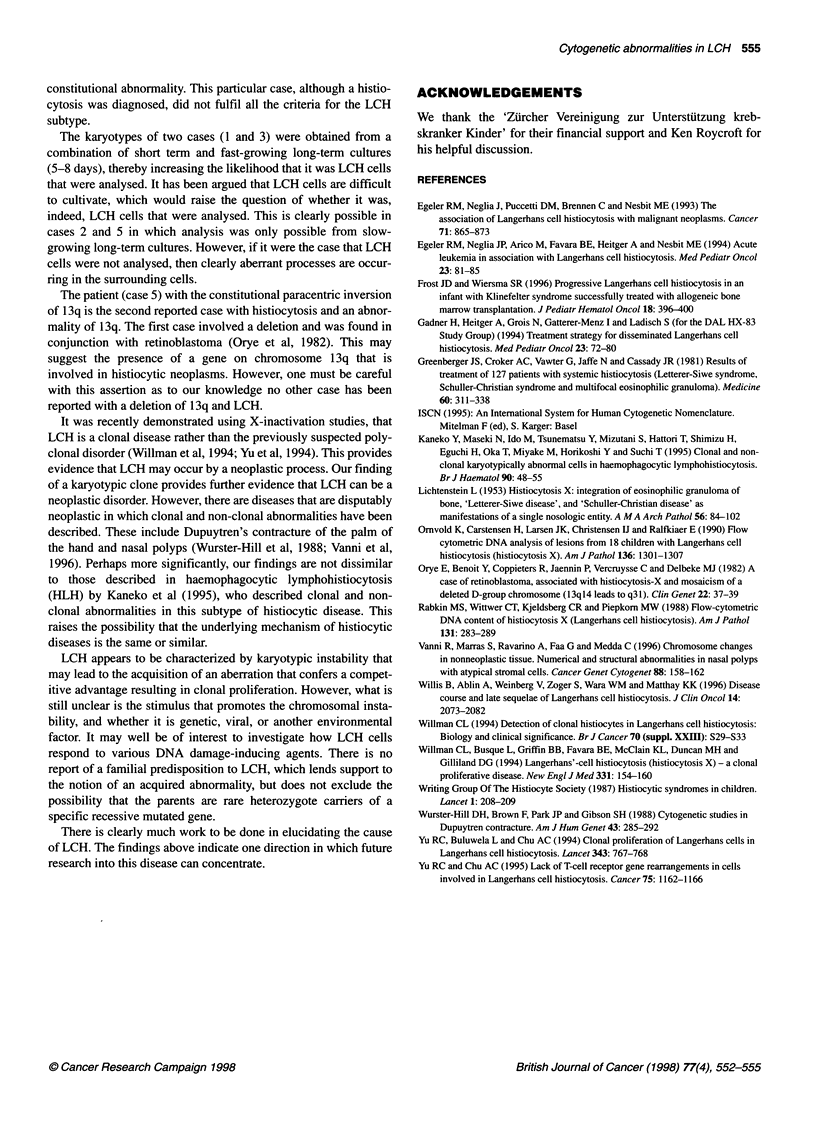# Cytogenetic abnormalities in Langerhans cell histiocytosis.

**DOI:** 10.1038/bjc.1998.89

**Published:** 1998-02

**Authors:** D. R. Betts, K. E. Leibundgut, A. Feldges, H. J. PlÃ¼ss, F. K. Niggli

**Affiliations:** Department of Oncology, University Children's Hospital, Zurich, Switzerland.

## Abstract

**Images:**


British Journal of Cancer (1998) 77(4), 552–555
© 1998 Cancer Research Campaign

### Cytogenetic abnormalities in Langerhans cell histiocytosis

**DR Betts[1], KE Leibundgut[2], A Feldges[3], HJ Plüss[1] and FK Niggli[1]**

[1]Department of Oncology, University Children's Hospital, CH-8032 Zurich; [2]Department of Oncology, University Children's Hospital, CH-3010 Berne; [3]Department of Oncology, Children's Hospital, CH-9006 St Gallen, Switzerland

**Summary** We present the cytogenetic investigations of five histiocytic tumour lesions from children. In four cases there was a confirmed diagnosis of Langerhans cell histiocytosis (LCH) and one case of histiocytosis that did not fulfil all the criteria for true LCH. All five cases showed cytogenetic abnormalities, including the first report of an abnormal clone in LCH. The clone showed a t(7;12)(q11.2;p13) translocation and was detected in only a small percentage of cells. This case and a further three also contained non-clonal abnormalities and an increase in chromosome breakage. The fifth case, the only one in which no acquired abnormalities were seen, had a constitutional paracentric inversion of chromosome 13q.

**Keywords:** cytogenetics; Langerhans cell histiocytosis; clonal abnormalities

Langerhans cell histiocytosis (LCH) is a histiocytic proliferative disorder, formally called histiocytosis X by Lichtenstein in 1953 to integrate eosinophilic granuloma, Hand–Schüller–Christian disease, and Letterer–Siwe disease under a single nosologic entity. The aetiology and pathogenesis of the disease are still poorly understood. However, the criteria for the diagnosis and clinical classification of LCH has now been clearly defined by the Writing Group of the Histiocyte Society (1987). The disease has a clinical heterogeneity ranging from a potentially lethal leukaemia-like disorder, which primarily affects infants, to solitary lytic bone lesions. The intermediate forms of the disease are characterized by skin and bone lesions, varying forms of organ dysfunction, diabetes insipidus and a chronic indolent course. In all forms of LCH, the histopathological lesions are typically similar and are characterized by CD1a antigen positive histiocytes and the presence of Birbeck granules. Positive immunohistochemical staining for the S100 protein is frequent. It has been stated recently that despite a good survival rate, many LCH patients will have either further disease dissemination or late sequelae (Willis et al, 1996).

It was reported by Greenberger et al (1981) that in long-term survivors there was a 5% incidence of malignancy. It has been suggested that there is an association between LCH and an increased frequency of malignancy occurring (Egeler et al, 1993). This includes a high incidence of leukaemia that follows two distinct patterns (Egeler et al, 1994): acute lymphoblastic leukaemia tending to precede LCH, whereas acute myeloid leukaemia (AML) follows. The occurrence of AML may in some part be related to the treatment of LCH with VP16. However, in the study of Gadner et al (1994) with a cohort of 106 patients, no second malignancies were found within a follow-up of 4–8 years.

*Received 4 March 1997*
*Revised 16 July 1997*
*Accepted 23 July 1997*

*Correspondence to:* David Betts, Onkologie Labor, Kinderspital Zürich, Steinwiesstrasse 75, CH-8032 Zürich, Switzerland

It has long been considered that LCH is a reactive disorder of immune regulation rather than a neoplastic process. However, this idea has been challenged recently by the demonstration that LCH is a clonal disease (Yu et al, 1994; Willman et al, 1994). Nonetheless, there is still a lack of other evidence to support a neoplastic process. One further proof for clonality would be the evidence of T-cell rearrangements, but none were found by Yu and Chu (1995). Tumours are typically characterized by cytogenetic abnormalities and, in many instances, DNA aneuploidy. To date, only a small number of LCH lesions have been described with an abnormal DNA index (Rabkin et al, 1988; Ornvold et al, 1990), and it has been stated that there is typically a failure to obtain metaphases and to detect karyotypic abnormalities in lesional cells (Willman, 1994).

We present five cases of histiocytosis in which karyotypic analysis revealed either an abnormal clone, non-clonal abnormalities or a constitutional abnormality.

### PATIENTS AND METHODS

The five children represent a consecutive series of patients with a histiocytosis, in which material was provided for cytogenetic analysis from different hospitals. All cases were analysed at diagnosis, with the exception of case 2, which was investigated 1 year after diagnosis. Microscopic evaluation of the lesion was clearly compatible with a LCH (class I histiocytosis) in four out of five cases. The fifth case had a clinical presentation typical for LCH but did not fulfil all the histological criteria.

Patient 1, a 2 and 3/12-year-old boy who presented with a soft-tissue tumour (4 × 4 cm) on the left frontotemporal side of his head. A magnetic resonance imaging (MRI) scan revealed bony destruction under the tumour mass. The tumour was grossly resected and histology showed a typical eosinophilic granuloma. Immunohistochemistry was positive for CD1a antigen and S100 protein. Further investigations revealed no additional bony or other organ lesions. The boy remains well 27 months after diagnosis.

**Table 1** Karyotypic results of the five cases

| Case | Source of cells | Time in culture (days) | Karyotype | Non-clonal abnormalities |
|---|---|---|---|---|
| 1 | Le | 1–8 | 46,XY[27] | add(6)(q25)/ del(7)(p1?)/ del(7)(q22)/ add(9)(q22)/ –16,+2mar/ –C,+mar |
| 2 | Le | 20 | 46,XY[43] | der(11)add(11)(p11.2)add(11)(q22),dic(14;?)(p11;?), –15,–18,+mar |
| | BM | 1 | 46,XY[50] | |
| 3 | Le | 1–5 | 46,XY,t(7;12)(q11.2;p13)[2]/46,XY[45] | add(1)(q12),–16,–17,+der(?)t(?;17)(?;q11.2),+mar/ t(2;4)(p21;q33),add(13)(p11.2)/ –11,–16,–22,+mar |
| 4 | Le | 6 | 46,XY[10] | del(8)(p12),–15,–20,+mar/ der(19)?t(9;19)(q12;q13) |
| 5 | Le | 20 | 46,XY,inv(13)(q21q33)c[10] | |
| | BM | 1–3 | 46,XY,inv(13)(q21q33)c[20] | |
| | PB | 3 | 46,XY,inv(13)(q21.2q33)mat | |

Le, histiocytic lesion; BM, bone marrow; PB, peripheral blood.

**Figure 1** A karyotype of a non-clonal abnormal metaphase from case 3, 46,XY,t(2;4)(p21;q33),add(13)(p11.2)

Patient 2 was diagnosed as a LCH at the age of 1 year with multiple organ involvement including skin, intestinal tract and later bone marrow. All the lesions showed eosinophilic cells and histiocytic elements that stained positive for CD1a antigen and S100 protein. He was treated with chemotherapy but suffered multiple relapses. To control his disease he finally underwent an allogeneic bone marrow transplantation 16 months after diagnosis and achieved a complete remission in which he remains 18 months after transplantation.

Patient 3, a 15-year-old boy who presented initially with a torticollis and later developed a decreased mobility of his left shoulder. A radiograph revealed a lytic lesion in the cervical vertebral body C5 and a soft tissue component comprising the nerve roots C4/C5. A decompression was performed and microscopic examination

© Cancer Research Campaign 1998 

**Figure 2** Normal chromosome 13 and inverted chromosome 13 from the peripheral blood of case 5 (breakpoints are arrowed)

showed an eosinophilic granuloma with CD1a antigen-positive and S100 protein-positive cells. Chemotherapy was introduced according to the international LCH II protocol and the patient achieved remission.

Patient 4 was diagnosed as LCH at the age of 9 years with a lytic lesion in his femur. After the biopsy the patient achieved a spontaneous remission. Histology showed a dense granulation tissue with eosinophilic granulocytes. The typical histiocytic elements revealed positivity for the immunohistochemical staining of the CD1a antigen and S100 protein.

Patient 5, a phenotypically normal boy who was born one week late after an uneventful pregnancy to unrelated parents. There was no reported family history of tumours. At the age of 5 months he developed a tumour on his frontal cranium, growing from the scalp to the meningeal membrane. The histology of the biopsy showed an eosinophilic granuloma but immunohistochemistry did not show CD1a antigen-positive cells or S100 protein positivity. A histiocytosis could be diagnosed but the biopsy did not fulfil the criteria for a true LCH. A juvenile xanthogranuloma (class II histiocytosis) could be the differential diagnosis. Owing to continued tumour growth after resection, treatment according to the international LCH II protocol was commenced.

Infiltrated tissue from all five cases was mechanically dissociated with scalpels to create material suitable for short- and long-term cultures. The cells were cultured in Nut.Mix F10 with Glutamax 1 (Gibco, Paisley, UK), supplemented with 15% fetal bovine serum and 1% penicillin–streptomycin. The short term cultures were harvested 16–72 h after induction of the culture. The seeded cultures were regularly monitored and the medium changed every three days, until sufficient growth was observed to facilitate cytogenetic analysis. The adherent cells were removed by trypsin/EDTA before harvesting according to recognized methods. Slides were made from fixed cell suspensions and, following ageing, were trypsin G-banded and Giemsa stained. The bone marrow samples from cases 2 and 5 and the phytohaemagglutinin (PHA)-stimulated peripheral blood investigation of case 5 and his parents were cultured by recognized methods. Cytogenetic analysis and interpretation was made according to ISCN 1995.

To establish if further evidence was present to indicate instability, analysis was performed on the banded preparations of cases 1–3 looking for spontaneous chromosomal breakage. The morphology of chromosomes in cases 4 and 5 was too poor to allow this analysis.

### RESULTS

A cytogenetic result derived from the histiocytic lesion was possible in all five cases (see Table 1), with analysis possible from short-term cultures in cases 1 and 3. In four cases (1–4) non-clonal acquired abnormalities were observed (Figure 1) and in one case (3) a low level clone was also present. The abnormal metaphases were either diploid or hypodiploid and unbalanced. Case 5 was found to have an inversion of chromosome 13 in the biopsy and bone marrow samples, which was subsequently proved to be constitutional (Figure 2). The breakpoints of the inversion could be defined as 13q21.2 and q33.

The cytogenetic investigation of the parents demonstrated that the 13q inversion was maternally inherited. The father had a normal 46,XY male karyotype.

The chromosome breakage study of cases 1–3 (see Table 2), revealed a breakage level typically higher than that seen in like cultures from other patients with neoplasms. The breakage seen was usually small chromatid breaks and the only break seen on more than one occasion was at 2q31. The characterized breaks were then compared with known fragile sites to determine if any pattern existed. It was found that 47% of breaks were located at known group 1 common fragile sites, and no bands associated with other fragile site types were affected. The figure of 47% is higher than would be expected by chance at the 400-band level, although it must be considered that if sub-band determination was possible this figure is likely to be lower.

### DISCUSSION

The description of chromosomal abnormalities in LCH is rare and is limited to case reports of patients with constitutional abnormalities (Orye et al, 1982; Frost and Wiersma, 1996). We believe this is the first report that describes cytogenetic abnormalities in the LCH lesions. Four cases were found to have either clonal or non-clonal abnormalities in 2–18% of analysable metaphases. A fifth case had no detectable acquired abnormalities in the lesion but did have a

**Table 2** The number of metaphases containing acquired chromosomal aberrations in the analysis of histiocytic lesions

| Case | Total metaphases examined | Metaphases with structural abnormalities | Metaphases with breaks | Metaphases containing an abnormality (%) |
|---|---|---|---|---|
| 1 | 33 | 6 | 4 | 27 |
| 2 | 44 | 1 | 6 | 16 |
| 3 | 50 | 5 | 11 | 30 |
| 4 | 12 | 2 | ND | 17 |
| 5 | 10 | 0 | ND | 0 |

ND, not done.

© Cancer Research Campaign 1998

constitutional abnormality. This particular case, although a histiocytosis was diagnosed, did not fulfil all the criteria for the LCH subtype.

The karyotypes of two cases (1 and 3) were obtained from a combination of short term and fast-growing long-term cultures (5–8 days), thereby increasing the likelihood that it was LCH cells that were analysed. It has been argued that LCH cells are difficult to cultivate, which would raise the question of whether it was, indeed, LCH cells that were analysed. This is clearly possible in cases 2 and 5 in which analysis was only possible from slow-growing long-term cultures. However, if it were the case that LCH cells were not analysed, then clearly aberrant processes are occurring in the surrounding cells.

The patient (case 5) with the constitutional paracentric inversion of 13q is the second reported case with histiocytosis and an abnormality of 13q. The first case involved a deletion and was found in conjunction with retinoblastoma (Orye et al, 1982). This may suggest the presence of a gene on chromosome 13q that is involved in histiocytic neoplasms. However, one must be careful with this assertion as to our knowledge no other case has been reported with a deletion of 13q and LCH.

It was recently demonstrated using X-inactivation studies, that LCH is a clonal disease rather than the previously suspected polyclonal disorder (Willman et al, 1994; Yu et al, 1994). This provides evidence that LCH may occur by a neoplastic process. Our finding of a karyotypic clone provides further evidence that LCH can be a neoplastic disorder. However, there are diseases that are disputably neoplastic in which clonal and non-clonal abnormalities have been described. These include Dupuytren's contracture of the palm of the hand and nasal polyps (Wurster-Hill et al, 1988; Vanni et al, 1996). Perhaps more significantly, our findings are not dissimilar to those described in haemophagocytic lymphohistiocytosis (HLH) by Kaneko et al (1995), who described clonal and non-clonal abnormalities in this subtype of histiocytic disease. This raises the possibility that the underlying mechanism of histiocytic diseases is the same or similar.

LCH appears to be characterized by karyotypic instability that may lead to the acquisition of an aberration that confers a competitive advantage resulting in clonal proliferation. However, what is still unclear is the stimulus that promotes the chromosomal instability, and whether it is genetic, viral, or another environmental factor. It may well be of interest to investigate how LCH cells respond to various DNA damage-inducing agents. There is no report of a familial predisposition to LCH, which lends support to the notion of an acquired abnormality, but does not exclude the possibility that the parents are rare heterozygote carriers of a specific recessive mutated gene.

There is clearly much work to be done in elucidating the cause of LCH. The findings above indicate one direction in which future research into this disease can concentrate.

### ACKNOWLEDGEMENTS

We thank the 'Zürcher Vereinigung zur Unterstützung krebskranker Kinder' for their financial support and Ken Roycroft for his helpful discussion.

### REFERENCES

Egeler RM, Neglia J, Puccetti DM, Brennen C and Nesbit ME (1993) The association of Langerhans cell histiocytosis with malignant neoplasms. *Cancer* **71**: 865–873

Egeler RM, Neglia JP, Arico M, Favara BE, Heitger A and Nesbit ME (1994) Acute leukemia in association with Langerhans cell histiocytosis. *Med Pediatr Oncol* **23**: 81–85

Frost JD and Wiersma SR (1996) Progressive Langerhans cell histiocytosis in an infant with Klinefelter syndrome successfully treated with allogeneic bone marrow transplantation. *J Pediatr Hematol Oncol* **18**: 396–400

Gadner H, Heitger A, Grois N, Gatterer-Menz I and Ladisch S (for the DAL HX-83 Study Group) (1994) Treatment strategy for disseminated Langerhans cell histiocytosis. *Med Pediatr Oncol* **23**: 72–80

Greenberger JS, Croker AC, Vawter G, Jaffe N and Cassady JR (1981) Results of treatment of 127 patients with systemic histiocytosis (Letterer-Siwe syndrome, Schuller-Christian syndrome and multifocal eosinophilic granuloma). *Medicine* **60**: 311–338

ISCN (1995): An International System for Human Cytogenetic Nomenclature. Mitelman F (ed), S. Karger: Basel

Kaneko Y, Maseki N, Ido M, Tsunematsu Y, Mizutani S, Hattori T, Shimizu H, Eguchi H, Oka T, Miyake M, Horikoshi Y and Suchi T (1995) Clonal and non-clonal karyotypically abnormal cells in haemophagocytic lymphohistiocytosis. *Br J Haematol* **90**: 48–55

Lichtenstein L (1953) Histiocytosis X: integration of eosinophilic granuloma of bone, 'Letterer-Siwe disease', and 'Schuller-Christian disease' as manifestations of a single nosologic entity. *A M A Arch Pathol* **56**: 84–102

Ornvold K, Carstensen H, Larsen JK, Christensen IJ and Ralfkiaer E (1990) Flow cytometric DNA analysis of lesions from 18 children with Langerhans cell histiocytosis (histiocytosis X). *Am J Pathol* **136**: 1301–1307

Orye E, Benoit Y, Coppieters R, Jaennin P, Vercruysse C and Delbeke MJ (1982) A case of retinoblastoma, associated with histiocytosis-X and mosaicism of a deleted D-group chromosome (13q14 leads to q31). *Clin Genet* **22**: 37–39

Rabkin MS, Wittwer CT, Kjeldsberg CR and Piepkorn MW (1988) Flow-cytometric DNA content of histiocytosis X (Langerhans cell histiocytosis). *Am J Pathol* **131**: 283–289

Vanni R, Marras S, Ravarino A, Faa G and Medda C (1996) Chromosome changes in nonneoplastic tissue. Numerical and structural abnormalities in nasal polyps with atypical stromal cells. *Cancer Genet Cytogenet* **88**: 158–162

Willis B, Ablin A, Weinberg V, Zoger S, Wara WM and Matthay KK (1996) Disease course and late sequelae of Langerhans cell histiocytosis. *J Clin Oncol* **14**: 2073–2082

Willman CL (1994) Detection of clonal histiocytes in Langerhans cell histiocytosis: Biology and clinical significance. *Br J Cancer* **70 (suppl. XXIII)**: S29–S33

Willman CL, Busque L, Griffin BB, Favara BE, McClain KL, Duncan MH and Gilliland DG (1994) Langerhans'-cell histiocytosis (histiocytosis X) – a clonal proliferative disease. *New Engl J Med* **331**: 154–160

Writing Group Of The Histiocyte Society (1987) Histiocytic syndromes in children. *Lancet* **1**: 208–209

Wurster-Hill DH, Brown F, Park JP and Gibson SH (1988) Cytogenetic studies in Dupuytren contracture. *Am J Hum Genet* **43**: 285–292

Yu RC, Buluwela L and Chu AC (1994) Clonal proliferation of Langerhans cells in Langerhans cell histiocytosis. *Lancet* **343**: 767–768

Yu RC and Chu AC (1995) Lack of T-cell receptor gene rearrangements in cells involved in Langerhans cell histiocytosis. *Cancer* **75**: 1162–1166

© Cancer Research Campaign 1998